# Can modern infrared analyzers replace gas chromatography to measure anesthetic vapor concentrations?

**DOI:** 10.1186/1471-2253-8-2

**Published:** 2008-02-08

**Authors:** Jan FA Hendrickx, Hendrikus JM Lemmens, Rik Carette, Andre M De Wolf, Lawrence J Saidman

**Affiliations:** 1 Department of Anesthesia, Stanford University School of Medicine, Stanford, California, USA; 2 Anesthesiologist, Department of Anesthesia, OLV Hospital, Aalst, Belgium; 3 Department of Anesthesiology, Feinberg School of Medicine, Northwestern University, Chicago, Illinois, USA

## Abstract

**Background:**

Gas chromatography (GC) has often been considered the most accurate method to measure the concentration of inhaled anesthetic vapors. However, infrared (IR) gas analysis has become the clinically preferred monitoring technique because it provides continuous data, is less expensive and more practical, and is readily available. We examined the accuracy of a modern IR analyzer (M-CAiOV compact gas IR analyzer (General Electric, Helsinki, Finland) by comparing its performance with GC.

**Methods:**

To examine linearity, we analyzed 3 different concentrations of 3 different agents in O_2_: 0.3, 0.7, and 1.2% isoflurane; 0.5, 1, and 2% sevoflurane; and 1, 3, and 6% desflurane. To examine the effect of carrier gas composition, we prepared mixtures of 1% isoflurane, 1 or 2% sevoflurane, or 6% desflurane in 100% O_2 _(= O_2 _group); 30%O_2_+ 70%N_2_O (= N_2_O group), 28%O_2 _+ 66%N_2_O + 5%CO_2 _(= CO_2 _group), or air. To examine consistency between analyzers, four different M-CAiOV analyzers were tested.

**Results:**

The IR analyzer response in O_2 _is linear over the concentration range studied: IR isoflurane % = -0.0256 + (1.006 * GC %), R = 0.998; IR sevoflurane % = -0.008 + (0.946 * GC %), R = 0.993; and IR desflurane % = 0.256 + (0.919 * GC %), R = 0.998. The deviation from GC calculated as (100*(IR-GC)/GC), in %) ranged from -11 to 11% for the medium and higher concentrations, and from -20 to +20% for the lowest concentrations. No carrier gas effect could be detected. Individual modules differed in their accuracy (p = 0.004), with differences between analyzers mounting up to 12% of the medium and highest concentrations and up to 25% of the lowest agent concentrations.

**Conclusion:**

M-CAiOV compact gas IR analyzers are well compensated for carrier gas cross-sensitivity and are linear over the range of concentrations studied. IR and GC cannot be used interchangeably, because the deviations between GC and IR mount up to ± 20%, and because individual analyzers differ unpredictably in their performance.

## Background

Gas chromatography (GC) has often been considered the most accurate method to measure the concentration of potent inhaled anesthetics (further referred to as "anesthetic vapors"). Infrared (IR) gas analysis provides continuous data and is more readily available clinically, less expensive and more practical, but needs complex calibration and compensation procedures to minimize or eliminate the effects of overlapping IR absorption spectra of anesthetic vapors, H_2_O, N_2_O, and CO_2 _and those of "collision broadening" or "pressure broadening" [1]. The effect of H_2_O has "virtually been eliminated" by the use of Nafion™ tubing [2,3], except possibly for the older agent halothane [4]. The effect of N_2_O is small, being reported as absent [4], less than 0.01 [5] or -.03 volume vol % with isoflurane and enflurane [2]. The effect of CO_2 _is absent [2] or less than +/- 0.01 vol% [3,5]. Most authors consider the response of these older IR analyzers to be sufficiently accurate and linear for clinical purposes, but some departure from linearity was found. The Datex Normac "under-read" enflurane and isoflurane concentrations in the low range and "over-read" them at the higher range, with the greatest error 6% of reading [2]. The Datex Capnomac Ultima under-predicted the concentrations of the agents by 10 to 12% [3].

While the issue of cross-sensitivity of other gases on IR analysis of anesthetic vapors has been resolved, extrapolating the accuracy of these older monitors to modern gas analyzers is complex. First, none of previous studies rigorously examined the performance with sevoflurane or desflurane. Second, in the manufacturing process, each module and each individual filter is compensated for cross gas effects. While this would suggest that analyzers might differ in their performance because the degree of compensation might differ, the issue has not been addressed in older studies because they only examined one analyzer. To assess instrumental variances, data were therefore obtained from 4 M-CAiOV compact IR gas analyzers (General Electric [Datex-Ohmeda], Helsinki, Finland)[6]. Finally, modern IR analyzers use a different part of the IR spectrum. The M-CAiOV unit uses the 8 to 9 μm range to eliminate the effect of CO_2 _and to minimize the effect of N_2_O. The use of 5 wavelengths between 8 to 9 μm allows automated agent detection and a correction factor for the effect of N_2_O for each of these 5 wavelengths. We were particularly interested in the performance of the M-CAiOV unit [6] because its ability to measure vapor concentrations accurately in clinical studies has not been determined [7]. We therefore studied the performance of 4 units, and examined whether IR accuracy approaches that of GC.

## Methods

The analytic accuracy of the IR analyzer in the M-CAiOV compact multi-gas analyzer for isoflurane, desflurane, and sevoflurane was determined by having gas mixtures with different concentrations of agent and carrier gases analyzed by both IR and GC. For clinical relevancy, concentrations ranged from MACawake (the concentration that suppresses response to verbal command in 50% of patients after equilibration between the brain and the end-expired concentration of the vapor) to MAC (the concentration that suppresses motor response to noxious stimulation in 50% of patients after equilibration between the brain and the end-expired concentration of the vapor). We prepared 3 different concentrations for each agent in O_2_, further referred to as low, medium, and high agent concentration: 0.3, 0.7, and 1.2% isoflurane; 0.5, 1, and 2% sevoflurane; and 1, 3, and 6% desflurane. To examine a possible effect of carrier gas composition, we prepared mixtures of inhaled agents with clinically relevant concentrations – 1% isoflurane, 6% desflurane, or 1 or 2% sevoflurane in 100% O_2 _(= O_2 _group); 30%O_2_+70%N_2_O (= N_2_O group), 28%O_2_+66%N_2_O+5%CO_2 _(= CO_2 _group), and air (= air group). Data were obtained from 4 M-CAiOV units to assess instrumental variances.

All gas mixtures were prepared with a conventional anesthesia machine (ADU Delivery Unit by GE, Helsinki, Finland), and sampled from the common gas outlet. For each analyzer, test mixtures were sampled from the common gas outlet at different times; because vaporizer output (and thus the concentration of vapor at the common gas outlet) may differ slightly from moment to moment, test mixtures may differ slightly between analyzers. For the CO_2 _groups, 5 mL CO_2 _(from an E-cylinder) was added to a volume of 95 mL mixture of agent with 30%O_2 _+ 70%N_2_O, yielding approximately 5% CO_2 _in the final mixture. All samples were drawn into 100 mL glass syringes. To ensure adequate mixing of the gases, the mixtures were injected via a three-way stopcock at least 4 times into a second 100 mL glass syringe. Immediately after mixing, the first and last 10 mL were injected into the gas chromatograph; the mid portion, 80 mL, was sampled by the IR analyzer. When the GC peak height of the first and last 10 mL sample varied by more than 1 mm, the measurements were repeated. Over the entire course of the study, repeat measures were done on three occasions because operator error had caused gross errors.

A flame ionization detector GC (Gow-Mac 580; Gow-Mac, Bethlehem, PA) was used, equipped with a 4.57 m, 22-mm internal diameter column containing 10% SF-96 on WHP, 68/80 mesh, maintained at 43°C with an 8 mL/min N_2_carrier flow. The detector (at 122°C) received H_2 _at 20 mL/min and air at 200 mL/min. The GC was calibrated before and at intervals during each test using secondary (cylinder) calibration standards. The same calibration gas (one for each agent) was used throughout the study. The calibration gas comes out of a stainless steel cylinder which contains a known concentration of vapor; for each agent, a different cylinder was prepared. The mixture is prepared by creating a subatmospheric pressure in the cylinder. Next, an amount of liquid agent is aspirated into the cylinder. This amount is calculated such that after vaporization the calculated pressure in the cylinder will remain below the vapor pressure of the agent. The cylinder is then filled/pressurized with air. To mix its contents, the cylinder is rolled several times, and subsequently heated overnight by applying an external heat pad. The exact concentration of this secondary standard is determined by comparing it with a primary volumetric standard using gas chromatography. The primary standard is prepared by injecting a known exact amount of liquid agent (measured by weight or glass micropipette) in a glass Erlenmeyer of known volume, taking the effect of temperature into account. Using this primary standard, it was determined that the concentrations of isoflurane, sevoflurane, and desflurane in the tank was 0.744, 1.49, and 4.20%, respectively. Over the entire course of the study, the peak height with the same calibration gas for isoflurane (0.744%), sevoflurane (1.49%), and desflurane (4.20%) was 54.0 mm (standard deviation 1.1 mm) for isoflurane, 52.2 mm (standard deviation 2.0 mm) for sevoflurane, and 54.1 mm (standard deviation 1.4 mm) for desflurane; the coefficients of variation for isoflurane, sevoflurane, and desflurane therefore were 2.0, 3.8, and 2.6 % respectively. These calculations are based on 6 calibrations, 3 before and 3 after measuring each agent for each analyzer (total of 18 per analyzer).

The 4 multi-gas analyzers were calibrated using the appropriate calibration gas provided (General Electric [Datex-Ohmeda], Helsinki, Finland), and as recommended analyses were started no sooner than 30 min after inserting the module.

### Statistical analysis

Repeated injections of a given sample from a tank or flask containing known volumetric standards into a GC give values with a standard deviation of less than 2–3% (personal communication with Dr. Eger EI II, and corroborated by our own findings in this study – coefficients of variation for isoflurane, sevoflurane, and desflurane of 2.0, 3.8, and 2.6 % respectively). Because GC is a calibrated reference standard, it can be considered to be an accurate measure of the concentrations within the known limits of accuracy. IR is therefore compared directly to the GC measurements, using linear regression analysis to examine the linearity of the response. To examine the effect of carrier gas and whether analyzers differed in their performance, we used two way ANOVA (factors = carrier gas and analyzer) with the Holm-Sidak method.

## Results

**Figure 1:**
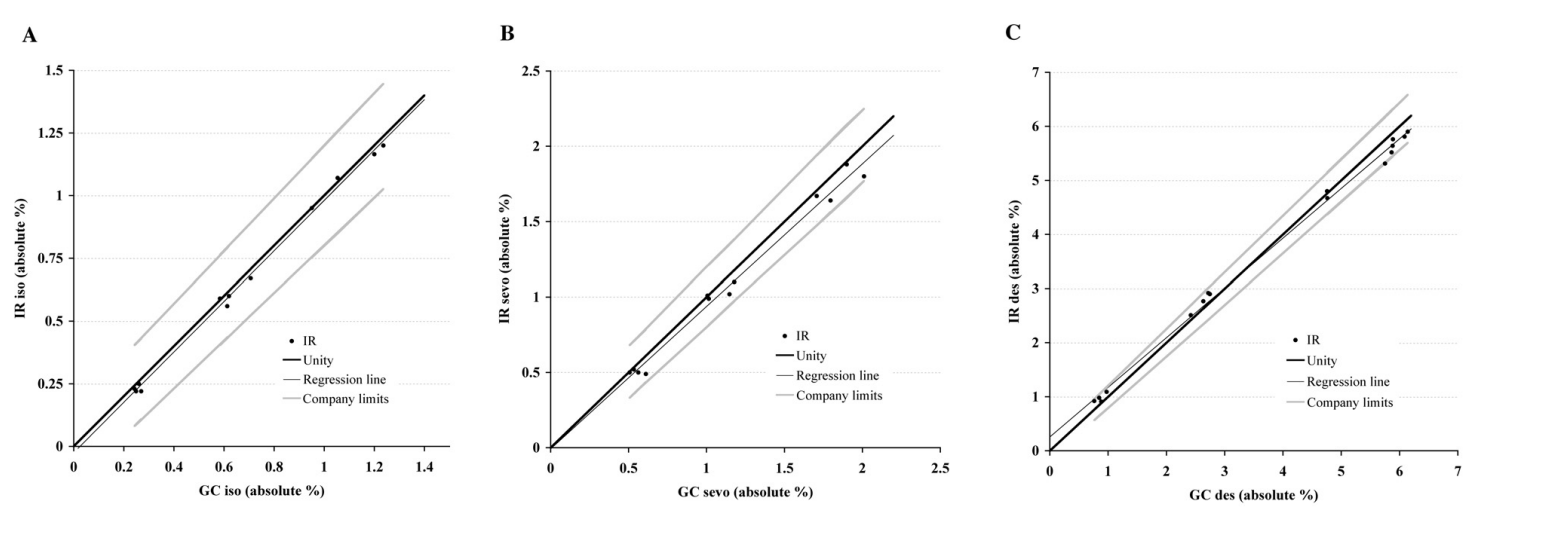
Linear regression plots comparing IR with GC analysis of isoflurane (A), sevoflurane (B), and desflurane (C) in O_2 _over a range of concentrations. Closed circles = individual data points; thick black line = line of unity; thin grey line = linear regression (see text for formulas), thick grey lines = upper and lower accuracy limits according to company. The performance of the four modules conformed to that specified by the company, +/- (0.15% + 5% of IR reading).

Linear regression results are presented in figure 1. The following mathematical expression described the response of the IR analyzer, with the standard error of the 2 linear regression parameters between square brackets: IR isoflurane % = -0.026 + (1.006 * GC %), R = 0.998, [0.014; 0.0185]; IR sevoflurane % = -0.008 + (0.946 * GC %), R = 0.993, [0.044; 0.034]; IR desflurane % = 0.256 + (0.919 * GC %), R = 0.998, [0.060; 0.014]. The deviation from GC calculated as (100* [IR-GC]/GC) for the medium and high concentrations ranged from -9 to 6% for isoflurane, from -11 to 5% for sevoflurane, and from -9 to 11% for desflurane. Deviation was more pronounced with the lower concentrations: from -18 to 2% for isoflurane, from -20 to 0% for sevoflurane, and from -8 to 21% for desflurane.

The difference between GC and IR analysis of volatile anesthetics was not affected by carrier gas (P = 0.942), but individual modules differed in their accuracy (p = 0.004), with the differences as great as 25% with the lowest agent concentrations and as great as 12% with the middle and highest concentrations (Table 1).

**Table 1:** Carrier gas effect.

		**O**_**2**_		**CO**_**2**_		**N**_**2**_**O**		**N**_**2**_**O+CO**_**2**_	
**Agent**	**Analyzer #**	**GC**	**IR**	**GC**	**IR**	**GC**	**IR**	**GC**	**IR**

**isoflurane**	**I**	1.24	1.20	1.18	1.15	1.19	1.15	1.22	1.20
	**II**	1.05	1.07	0.97	1.01	1.08	1.10	1.18	1.22
	**III**	0.95	0.95	0.88	0.86	1.24	1.30	1.11	1.12
	**IV**	1.20	1.17	0.85	0.81	1.14	1.06	1.08	1.00

**sevoflurane**	**I**	1.90	1.88	1.96	1.90	0.91	0.93	0.88	0.88
	**II**	2.01	1.80	1.08	0.99	1.05	0.98	0.98	0.89
	**III**	1.71	1.67	1.82	1.80	0.95	0.91	1.03	1.00
	**IV**	1.80	1.64	1.80	1.64	1.05	0.98	0.84	0.77

**desflurane**	**I**	6.14	5.90	5.00	5.00	5.95	6.03	5.71	5.40
	**II**	5.88	5.64	5.38	5.27	5.86	5.56	5.48	5.37
	**III**	4.76	4.80	6.12	5.96	6.76	6.47	6.02	5.54
	**IV**	5.75	5.31	5.29	5.00	5.85	5.40	5.65	5.30

Carrier gas composition did not affect IR analysis (P > 0.05), but individual modules within the carrier gas group differed in their accuracy (P = 0.004). GC = gas chromatography (measured value, % of atm); IR = infrared values (measured value, % of atm).

## Discussion

In this study, we confirm that IR analysis of anesthetic vapors by a polychromatic IR analyzer is not affected by carrier gas composition. The response by the IR analyzer is linear for the three agents over the concentration range tested (R > 0.99), but it tends to underestimate the concentrations of isoflurane and sevoflurane and those of desflurane at higher concentrations. The response differs unpredictably between modules.

Inhaled anesthetic vapors absorb IR, allowing their concentration to be measured using the law of Beer-Lambert. More specifically, they absorb IR at 3.3 μm and in the 8 to 9 μm range. Because of the proximity of a CO_2 _absorption band at 3.3 μm used by older analyzers, CO_2 _interfered with the measurement of anesthetic vapors. Even though the older analyzers compensated for this effect, the use of five different wavelengths in the 8 to 9 μm range eliminates this issue completely.

When N_2_O is present, overlapping absorption spectra complicate the measurement of anesthetics agents. The effect is reduced by the use of the 8–9 μm range, but there still is a need for cross compensation caused by the absorption of N_2_O at the wavelengths used for anesthetic agents. The magnitude of the effect of N_2_O on anesthetic agent measurements depends on the individual characteristics of the optical filters in the IR-sensor of a module. In the manufacturing process, each module and each individual filter is compensated for cross gas effects with N_2_O (personal communication with K. Karlsson, GE Healthcare). Interactions with N_2_O are tested with anesthetic agents, and the modules are then compensated for this effect. The compensation coefficients are stored in the EEPROM-memory of each module. After this compensation, the error caused by 79% N_2_O to the zero point of the anesthetic gas measurements must be smaller than +/- 0.1% halothane in order to have the module accepted from this calibration phase. Because the M-CAiOV module measures N_2_O simultaneously with anesthetic agent, it is possible to use a real time compensation for N_2_O in the calculation of the anesthetic agent concentration: instead of assuming a fixed N_2_O-concentration, the compensation uses the measured real time value of N_2_O as the input value. The complex calibrations and compensations in commercial sensors are proprietary, but they appear to be effective since we could not document an effect of N_2_O on the IR analysis of anesthetic vapors.

The effect of H_2_O vapor is minimized by equalizing the H_2_O vapor pressure in the sample to that in the atmosphere by using Nafion™ tubing[8]. The technology was already incorporated in older IR analyzers, and found to be effective [2,3]. Nafion™ is a copolymer of tetrafluoroethylene (Teflon^®^) and perfluoro-3,6-dioxa-4-methyl-7-octene-sulfonic acid). Sulfonic acid (-SO3H) has a high water-of-hydration, absorbing up to 13 molecules of H_2_O for every sulfonic acid group in the polymer. Unlike micro-porous membrane permeation, which transfers water through a relatively slow diffusion process, Nafion™ removes water by absorption as water-of-hydration, a first order kinetic reaction that equilibrates within milliseconds. H_2_O binds to the sulfonic acid in Nafion™ and will readily permeate through the polymer, thus equalizing the humidity of the gas going into the gas sensors with that of the ambient air. This is necessary for two reasons. First, calibration gas is dry, while patient samples have 100% relative humidity. Calibrating the sensor with dry gas and measuring saturated gas would cause an additional error to the measurements. Secondly, the sample gas coming from the patient circuit needs to pass the Nafion tube so that the humidity equals the ambient humidity before being analyzed. The gas concentrations are thus measured at the prevailing ambient humidity. If the ambient humidity varies very much between the calibration and the actual monitoring, there will be an additional error component caused by H_2_O.

What are the implications of our findings? Effects of cross-sensitivity by different gases are virtually absent, a finding we confirmed for CO_2_, N_2_O, N_2_, and O_2_. The performance of the four modules conformed to that specified by the company, +/- (0.15% + 5% of IR reading) (figure 1). The measurement accuracy of all tested modules is better than that required by the ISO 21647 standard for essential performance of respiratory gas monitors (ISO21647:2004(E)). Of the 4 analyzers we tested, IR slightly underestimates most anesthetic vapor concentrations, possibly causing the anesthesiologist to administer a slightly greater concentration. This may be more clinically relevant at the lower concentration range. At high concentrations, well above MAC awake and even MAC, the patient is adequately anesthetized and the dose given becomes a titration against vital sign fluctuations. At low concentrations however, in the MACawake range, one is more concerned about the potential for recall, especially in patients who cannot sustain hemodynamic stability in the presence of anesthetic vapors. A measurement that is lower than the actual concentration may lead the clinician to increase the concentration unnecessarily and/or use a vasopressor to maintain adequate blood pressure for the given depth of anesthesia. An error on the high side may lead to inadequate vapor delivery to prevent recall.

Can IR gas analysis be used for clinical research? Even though the use of different IR absorption bands by the M-CAiOV compact multi-gas analyzer has allowed automated agent detection and may technically have facilitated the compensation for cross-sensitivity between anesthetic vapors and other gases, it has not improved accuracy of vapor analysis beyond that of existing, older IR analyzers. Deviations from GC calculated as (100* [IR-GC]/GC) range from -11% to 11% around 0.5 MAC values, and higher still at lower concentrations. We also found that individual modules differ unpredictably in their accuracy, and that, even though the response is linear, some additional gain (sevoflurane) and offset (isoflurane) control or both (desflurane) may be needed. Finally, small effects of carrier gases can still be present (personal communication with K. Karlsson, GE Healthcare). A recent study by Peyton suggested that the accuracy and precision of measurement of volatile anesthetic gas partial pressures in blood by a double headspace equilibration technique, using a clinical infrared gas analyzer, were comparable to that achieved by previous studies using gas chromatography [9]. However, only one gas analyzer was examined. Because our study demonstrates difference in performance between individual units, our study suggests that GC remains the method of choice to measure absolute concentrations. An alternative interpretation of Peyton's findings might be that IR analysis could be used if the performance characteristics of the individual IR analyzer are well documented. IR analysis can certainly still be used if the clinically significant difference sought between groups would be larger than the possible error (approximately 10% of the displayed value) of the method. Any inaccuracies are less likely to have implications for studies that report F_A_/F_I _(inspired over end-expired concentrations), because any error should affect F_A _and F_I _almost proportionally. If a particular analyzer would be over-estimating the "true" concentration by 10% for whatever reason, the ratio of F_A_/F_I _would still be accurate: F_A_/F_I _= (F_A_*1.1)/(F_I_*1.1).

## Conclusion

In summary, the use of different IR absorption bands by the M-CAiOV compact multi-gas analyzer (General Electric) has allowed automated agent detection and may technically have facilitated the compensation for cross-sensitivity between anesthetic vapors and other gases, but has not improved accuracy of vapor analysis beyond that of older IR analyzers. IR and GC cannot be used interchangeably, because the deviations between GC and IR mount up to ± 20%, and because individual analyzers differ unpredictably in their performance.

## Competing interests

The author(s) declare that they have no competing interests.

## Authors' contributions

JH, HL, ADW, and LJS conceived the concept; HL and LJS provided the equipment and taught the analytical techniques; JH acquired the data; all authors helped prepare the manuscript, and have read and approved the final manuscript.

## Pre-publication history

The pre-publication history for this paper can be accessed here:


